# Integrating social determinants of health screening and referral during routine emergency department care: evaluation of reach and implementation challenges

**DOI:** 10.1186/s43058-021-00212-y

**Published:** 2021-10-07

**Authors:** Andrea S. Wallace, Brenda L. Luther, Shawna M. Sisler, Bob Wong, Jia-Wen Guo

**Affiliations:** 1 College of Nursing, University of Utah, 10 South 2000 East, Salt Lake City, UT 84112-5880 USA; 2 Department of Population Health Sciences, University of Utah School of Medicine, Salt Lake City, USA

**Keywords:** Social determinants of health, Socioeconomic factors, Emergency service, Referral and consultation, Health equity

## Abstract

**Background:**

Despite the importance of social determinants in health outcomes, little is known about the best practices for screening and referral during clinical encounters. This study aimed to implement universal social needs screening and community service referrals in an academic emergency department (ED), evaluating for feasibility, reach, and stakeholder perspectives.

**Methods:**

Between January 2019 and February 2020, ED registration staff screened patients for social needs using a 10-item, low-literacy, English-Spanish screener on touchscreens that generated automatic referrals to community service outreach specialists and data linkages. The RE-AIM framework, specifically the constructs of reach and adoption, guided the evaluation. Reach was estimated through a number of approaches, completed screenings, and receipt of community service referrals. Adoption was addressed qualitatively via content analysis and qualitative coding techniques from (1) meetings, clinical interactions, and semi-structured interviews with ED staff and (2) an iterative “engagement studio” with an advisory group composed of ED patients representing diverse communities.

**Results:**

Overall, 4608 participants were approached, and 61% completed the screener. The most common reason for non-completion was patient refusal (43%). Forty-seven percent of patients with completed screeners communicated one or more needs, 34% of whom agreed to follow-up by resource specialists. Of the 482 participants referred, 20% were reached by outreach specialists and referred to community agencies. Only 7% of patients completed the full process from screening to community service referral; older, male, non-White, and Hispanic patients were more likely to complete the referral process. Iterative staff (*n* = 8) observations and interviews demonstrated that, despite instruction for universal screening, patient presentation (e.g., appearance, insurance status) drove screening decisions. The staff communicated discomfort with, and questioned the usefulness of, screening. Patients (*n* = 10) communicated a desire for improved understanding of their unmet needs, but had concerns about stigmatization and privacy, and communicated how receptivity of screenings and outreach are influenced by the perceived sincerity of screening staff.

**Conclusions:**

Despite the limited time and technical barriers, few patients with social needs ultimately received service referrals. Perspectives of staff and patients suggest that social needs screening during clinical encounters should incorporate structure for facilitating patient-staff relatedness and competence, and address patient vulnerability by ensuring universal, private screenings with clear intent.

**Trial registration:**

ClinicalTrials.gov, NCT04630041.

**Supplementary Information:**

The online version contains supplementary material available at 10.1186/s43058-021-00212-y.

Contributions to the literature

- Despite the limited evidence of technical or logistical barriers to universal social needs screening and referrals, few emergency department patients with social needs received community outreach.
- When screening for unmet social needs, healthcare systems need to overtly acknowledge the role of patient stigmatization based on presentation and insurance status.
- To decrease staff discomfort in asking questions about unmet social needs, interventions should be developed and tested to improve staff familiarity/comfort in delivering screening, improve understanding of how social needs have implications for care, and promote engagement between staff and patients.

## Background

Over the last decade, it has become widely accepted that social determinants of health (SDOH), or the conditions in which people live, learn, work, and play [1], determine the majority of individual health outcomes [1]. However, while addressing SDOH is seen as critical to improving health outcomes [2], concerns about the ethical, privacy, and practical implications of universal screening efforts have been raised [3]; there is scant research to help healthcare systems design and implement feasible and effective strategies for addressing SDOH.

Current research efforts have largely focused on evaluating the impact of SDOH interventions on health outcomes and costs. To date, most research in the area has been limited to program evaluations of clinical services such as case management [4, 5], with a small number of randomized trials demonstrating that intensive SDOH interventions result in little improvement in traditional measures of physical health, or health system costs [6]. As a result, investigators continue to apply increasingly complex methodologies to examine SDOH, identify multi-level interventions for addressing SDOH, and seek measures that are both clinically meaningful to patients and sensitive to change. This work falls in contrast to healthcare systems rapidly adopting screenings of more limited “social needs” (e.g., for housing, food, transportation), which has resulted in discussions about the nature and content of social needs screening instruments and strategies [7]. However, one common theme of this work in research and practice settings is that all efforts should be underscored by the importance of developing and implementing social needs screening in a way that is linked to substantive and actionable processes and services to meet patient needs after screening [8–10].

One yet-to-be applied approach for examining the social needs screening and subsequent referral processes is to place them in the larger landscape of behavior change interventions. Such frameworks clearly outline that the first step in intervention effectiveness is participant engagement, namely that interventions aiming to address SDOH need to be delivered in a way that is well-received by patients and, through measurable steps, lead to having needs met. To date, research focusing specifically on patient engagement in these SDOH-focused interventions has demonstrated that patients are generally receptive to social needs screening and referral processes being embedded within healthcare services. Even in situations when needs cannot be met, patients understand the connection of how meeting social needs can improve their health [11]. However, others have found that, even when screening positive for social needs, a majority of patients may not wish to engage in processes to have needs met. A recent study by Tong [12] studied 123 patients, 85% of whom screened positive for social needs, but only 3% wanted help with those needs. Similarly, in a preliminary study conducted by this authoring research team [13], only half (52%) of those with needs were receptive to community service outreach. Collectively, these findings suggest that a key issue for research is to identify factors impacting the reach of social needs screening and outreach [11, 13].

While best practices for health-related screening are broadly available [14], and there are networks available for meeting social needs with community-based resource outreach [15, 16], little is known about how to best develop and support patients’ ability to complete referrals for needed services [17]. Further, there is limited research related to staff engagement in social needs interventions and whether and how staff may impact intervention delivery and patient receptivity. As such, the purpose of this study was to fully implement a universal screening for SDOH, or unmet social needs, in emergency department (ED) care settings using existing personnel and resources and offer to connect patients to free outreach by community service providers. We evaluated for the reach and adoption of the intervention and focused particular attention on staff and patient perceptions acting as barriers to, and facilitators of, social needs screening and community service outreach.

## Social needs screening and referral in routine emergency department care

As the only place in the US healthcare system where patients cannot be turned away for inability to pay, EDs care for a disproportionate number of low-income and uninsured patients [18, 19]. The work described in this paper capitalized on engagement between clinician-investigators and social service providers during which it was discovered that ED patients were commonly referred to the United Way’s 2-1-1 (211) service by ED care management staff. The 211 service, part of a nationwide network supported by United Ways and other non-profits, provides a free-of-charge, comprehensive list of contact information for local resource providers who address common social needs (e.g., transportation, financial advice, food, and housing assistance). The 211 service described in this work—Utah 211—is staffed 24 h per day, 7 days per week by trained resource specialists with access to an information pool of over 10,000 services in Utah and the surrounding states. Resource specialists are subject to routine quality oversight and use HIPAA-compliant software to track service use, consumer demographics, reported needs, and consumer follow-up.

Our academic clinical-211 partnership developed the 10-item, low-literacy social needs screener used in this project, the Screener for Intensifying Community Referrals for Health (SINCERE), using the Psychometric and Pragmatic Evidence Rating Scale (PAPERS). Described in detail elsewhere [13, 20], the development and evaluation process addressed key pragmatic measures for implementation research aspects impacting implementation including acceptability, relative advantage over existing methods, ease of completion, compatibility, organizational activities, informing clinical or organizational decision-making, cost, language accessibility, and assessor burden (training) [21–23]. A particular focus during screener development was to also identify needs subject to follow-up and service referrals by 211 after ED discharge, and engaged community and clinical stakeholders to develop, adopt, refine, and iteratively test the process using existing resources in the ED setting. A recent analysis of over 4000 screenings demonstrated SINCERE’s sound psychometric qualities [20].

## Methods

### Design overview

The larger parent study was guided by the RE-AIM (Reach, Effectiveness, Adoption, Implementation, and Maintenance) framework to evaluate the implementation components critical to understanding potential population health impact [24]. The data and analyses described in this paper focus particular attention on reach (the number of individual patients benefitting from the screening and outreach service referrals) and adoption (receptiveness of patients and providers to engage in screening and outreach service referrals). A convergent mixed-methods design was applied, in which quantitative screening and qualitative staff and patient data were simultaneously collected, analyzed separately, and then merged to obtain a deeper understanding of the feasibility and acceptability of social needs screening and outreach service referrals [25]. All methods were reviewed and approved by the University of Utah’s Institutional Review Board.

### Social needs screening and referral process

This study was conducted with adult patients seen in the University of Utah University Hospital ED, a tertiary academic health sciences level I trauma center located in the Mountain West. In January 2019, the registration staff, who generally interact with patients for contact and insurance information after ED admission to a treatment room, were given detailed information about the intervention and nature of 211 outreach, were introduced to an appointed 211 information specialist, and were trained on how to administer and document the 10-item English-Spanish social needs screener on touchpads and the Research Electronic Data Capture (REDCap; [26, 27]) online system.

The staff were instructed on how to allow patients to either self- or verbally respond to the questions in English or Spanish using the touchscreens. While asked to complete the screening with all patients being admitted to the ED, the staff were asked to use their discretion and omit patients with cognitive impairment, trauma, language other than English or Spanish, or residents of skilled nursing facilities. Between January 2019 and April 2020, ED registration staff then administered the screener and referral information as part of their standard ED admission workflow.

Preliminary work established that, on average, patients are able to complete the 10 dichotomous yes-no social needs questions in less than 80 s [13]. Immediately following the screening questions, patients were asked to share their preferred contact information if they wished to be contacted by a Utah 211 information specialist within 48 h of ED discharge. As part of our referral process, patients were given a written (via the touchscreen screener) and verbal (via registration staff) introduction to Utah 211 (211utah.org) describing it as a free service providing referrals to low- and no-cost community resources for needs, such as transportation, food, housing, and medications. All patients were asked if they would like a referral regardless of whether they had social needs.

Throughout the implementation trial, patient responses were tracked in REDCap. Real-time contact and social need response information were shared with appointed 211 resource specialists for all ED patients wishing outreach and service referrals via the HIPAA-complaint REDCap system. Patient zip codes were shared to pre-emptively assist information specialists locate resources in patients’ geographic areas. Resource specialists then proceeded to conduct outreach via telephone, text, and/or email, according to patient preference. Resource specialists entered all relevant encounter information regarding contacts and service referrals into their HIPAA-complaint Mediaware database, linked via a unique identifier by which 211 encounter details were routinely imported into the REDCap database. Finally, patient demographic details were imported into REDCap from the UHealth Enterprise Data Warehouse.

### Stakeholder observations and interviews

We gathered data about the adoption of the social needs screening through (1) individual observations and interviews with registration staff in the ED setting and (2) an intensive, iterative semi-structured focus group in a community engagement laboratory setting with an ED patient advisory group.

#### Staff observations and interviews

In preliminary work [13], the staff communicated some resistance and skepticism whether the scope of their role should be expanded to incorporate the social needs screener. In order to better understand the context and perspectives of the staff, in-depth, iterative interviews and observations were conducted with 8 registration team members, representing approximately one-third of the staff engaged in screening. Data were collected during clinical interactions and staff meetings by study investigators external to the department; demographic characteristics of the staff members were not collected to ensure anonymity.

Interviews explored each individual’s approach to the delivery of the screenings to patients and families, as well as attitudes toward perceived clinical utility, patient value, and overall acceptability (see Additional file 1 for interview guide). All interviews were recorded and transcribed. Memos from each interview were also recorded after each interaction and synthesized. Content analysis and qualitative coding techniques identified themes, and the results were abductively analyzed through the lens relative to self-determination theory (see Additional files 2 and 3).

#### Patient focus group

A purposive sample of ED patients who had accessed care at a large urban tertiary ED in the Intermountain West during the last 12 months (*n* = 10) was recruited through established hospital community advisory groups to participate in an iterative “community engagement studio.” Community engagement studios are utilized in research as a medium to dynamically interact with community stakeholders in order to receive feedback regarding the planning, design, implementation, and dissemination of interventions [28] Participants were prepared for the engagement studio via a telephone conversation during which the social needs screener and purpose of the intervention were shared. Participants were then asked to discuss the screening questions and intervention with the diverse communities they represented.

The engagement studio discussion, moderated by an experienced engagement team and observed by the research team, occurred in a single event over 2 h to explore the strengths, weaknesses, opportunities, and challenges of social needs screening and referrals, as well as concerns brought up in staff interviews (e.g., stigmatization, contextualization of the screener). The discussion was guided by the tenant that each participant in the group serves as a representative of their community, and the group as a collective represents the diverse community within the greater patient population seeking care in the ED. This one-time focus group method was used to help highlight individual responses, as well a process to co-construct meaning as a group [29]. As such, the research team focused on transcriptions of the substantive content shared, as well as notes documenting the conversational dynamics [30].

### Analysis

#### Quantitative data

Using data collected from REDCap and 211 Mediaware, descriptive summary statistics (means, standard deviations, frequency counts, and percentages) were calculated using SPSS (V 27). Independent *t*-tests and Pearson chi-square were used to assess the relationship between demographic variables (age, gender, race, ethnicity, and insurance type) with self-reported social needs (yes/no) and receipt social service referrals through 211 resource specialists (yes/no).

#### Staff interviews and observations

Files and data were uploaded to Atlas.ti [31] for qualitative analysis. To identify major themes and categories within the data, a detailed content and thematic analysis strategy was employed, leveraging a dual-coding system and verified audit trail. All data are electronically stored in a HIPAA-compliant cloud server.

From the initial substantive coding of each registration staff interview, the categorical properties iteratively emerged by fine-tuning code frequency and code comparison into broader themes. In parallel, results were abductively analyzed through the lens relative to self-determination theory. The role of self-determination theory was chosen via pilot data from a quantitative companion study, testing the feasibility of the intervention. It seeks to leverage the critical roles that competence, relatedness, and autonomy play in assisting patients or staff to become self-determined and engaged with the intervention [32]. The text was analyzed for codes and categories to identify commonalities and differences in the responses between participants as well as describing attitudinal or behavioral responses of the participants (Additional file 2).

#### Patient focus group

The focus group and individual responses were recorded, transcribed verbatim, and iteratively coded using methods offered for descriptive thematic analysis [33, 34]. In addition, analysis was guided by the theory of reasoned action (TRA) to help discover individuals’ motivation and beliefs related to completing social needs screening, participating in outreach and service connections. TRA assumes the predictors of a behavior are a person’s actions and beliefs regarding behavior and attitude [35]. As such, TRA components of attitudes, norms, and perceived control were examined to explain the intention and beliefs and, as such, predict the behaviors of these participants.

## Results

See Table 1 for an overview of this study’s conceptual linkages to the RE-AIM constructs of Reach and Adoption, data sources, variables, and primary findings.

**Table 1:** RE-AIM constructs of reach and adoption, data sources, variables, and primary findings

Construct	Data source	Variables	Key findings
Reach	REDCap	Number of screenings/number of screenings completed with needs indicated	46.9% indicated having > one social needs
	211 Resource Specialist Database	Number of patients with needs/number referred to 211; number referred/number contacted and referred to agencies by 211	34.2% with needs who desired referrals 20.3% referred reached for community-based services
Adoption	REDCap	Reasons given by staff for not screening a patient	36% of approaches were not screened *43% due to patient refusal* *16% too sick/trauma*
	Patient Engagement Studio	Patient-identified benefits of, and barriers to, social needs screening and referral processes	Potential embarrassment “Would I answer? Yes, Maybe, No” Need for sincerity “I need to know YOU before I answer.” “I need to feel the person cares.” Vulnerability “These questions make me vulnerable.” “I wouldn’t want these in my permanent record.”
	Staff observation	Barriers and facilitators of screening during patient encounters	Use of professional “intuition” to decide who to screen *Decisions not to screen based on patient insurance or appearance, “profiling”* Screener as the “right” tool *Staff make the screen [their] own.* Perceptions of usefulness *It is about the staff trusting that the information is useful.*
	Staff interviews	Provider-identified benefits of, and barriers to, social needs screening and referral process	

### Reach of social needs screening and referral intervention

**Fig. 1:**
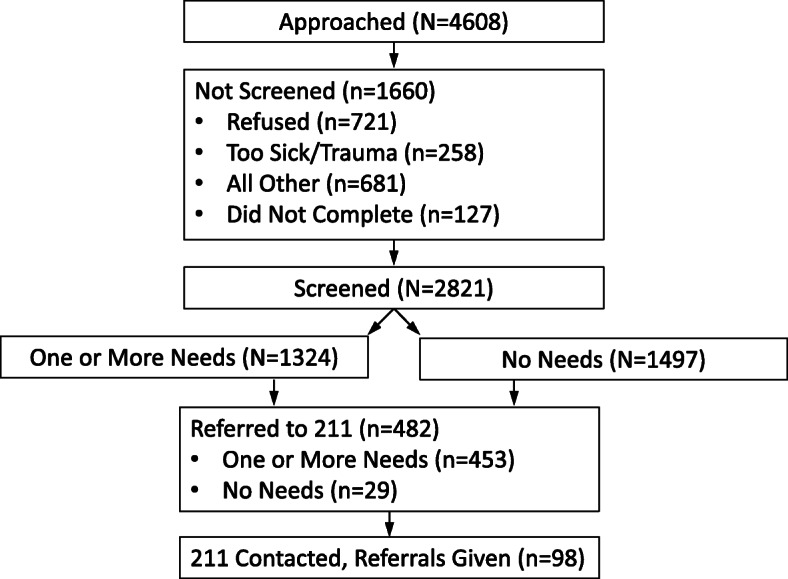
Retention of patients from approach to service referrals

Over 412 days (January 14, 2019, to February 29, 2020), 4608 patients were approached (Fig. 1). A total of 1660 (36%) were not screened (refused, 43%; too sick/trauma, 16%). A total of 2821 patients completed the screening. The average age of participants was 44.4 (17.8) years old (Table 2). The distribution of men and women was 45.0% vs. 55.0%, respectively. Of those completing screening, 14.2% were identified in their health record as Hispanic/Latino ethnicity, and 79% were listed as White/Caucasian for racial background. Only 2.4% (68) of participants answered the screener in Spanish.

**Table 2:** Participants expressing social needs vs. no social needs

	No needs	One or more needs	Total	*p* value
Characteristic	*N* = 1497	*N* = 1324	*N* = 2821^a^	
Age in years (SD)	47.1 (19.4)	41.2 (15.2)	44.4 (17.8)	< 0.001
Gender				0.136
Female	820 (56.4%)	679 (53.5%)	1499 (55.0%)	
Male	635 (43.6%)	590 (46.5%)	1225 (45.0%)	
Ethnic background				< 0.001
Chose not to disclose	12 (0.8%)	8 (0.6%)	20 (0.7%)	
Hispanic/Latino	158 (10.9%)	229 (18.1%)	387 (14.2%)	
Not Hispanic/Latino	1283 (88.3%)	1030 (81.3%)	2313 (85.0%)	
Race				< 0.001
American Indian and Alaska Native	9 (0.6%)	20 (1.6%)	29 (1.1%)	
Asian	41 (2.8%)	11 (0.9%)	52 (1.9%)	
Black or African American	35 (2.4%)	72 (5.7%)	107 (3.9%)	
Choose not to disclose	5 (0.3%)	7 (0.6%)	12 (0.4%)	
Native Hawaiian and Pacific Islander	25 (1.7%)	19 (1.5%)	44 (1.6%)	
Others	135 (9.3%)	198 (15.6%)	333 (12.2%)	
White or Caucasian	1203 (82.8%)	940 (74.2%)	2143 (78.8%)	

^a^Overall missing demographic data = 3.5%

Of the 2821 participants screened (Fig. 1), 1324 (46.9%) indicated having one or more social needs. Of the 1324 participants indicating having one or more social needs, 453 (34.2%) wished referral to 211. In addition, there were 29 participants that asked to be referred to 211 who indicated having no social needs. Of the 482 participants who were referred to 211, 98 (20.3%) were eventually reached by 211 information specialists and were given referrals to community-based agencies. The overall percentage of those with needs who were given referrals to community-based agencies was 7.4% (98/1324) or 3.5% of the total sample screened (98/2821).

In an effort to understand the demographic factors contributing to the differences in reported social needs, we compared the demographic characteristics (age, gender, race, and ethnicity) between the 1324 patients who indicated one or more needs and the 1497 patients who indicated no needs. The results (Table 2) show the patients who indicated one or more needs were significantly younger with 41.2 (15.2) years for those with needs vs. 47.1 (19.4) years without needs, *p* < 0.001. There was a higher percentage of Hispanic ethnicity (18.1% vs. 10.9%), *p* < 0.001, and a higher percentage of Black or African American (5.7% vs. 2.4%) and other racial backgrounds (15.6% vs. 9.3%), *p* < .001, for those with one or more social need. There were no gender differences, *p* = 0.136.

Next, to better understand intervention engagement, we compared the demographic characteristics between the 98 participants who received community service referrals vs. the 384 participants who asked for outreach but did not connect with the 211information specialist for community service referrals. The results (Table 3) indicate that patients who received referrals were older (46.5 (17.0) years vs. 42.3 (14.2) years, *p* = 0.029). Those who received service referrals were more likely to be of Hispanic ethnicity (32.3% vs. 20.6%), *p* = 0.042; have a non-White racial background (32.3% vs. 16.9%), *p* = 0.004; or be male (62.8% vs. 47.5%), *p* = 0.008. The average number of needs reported was not significantly different, with 4.7 (2.5) needs reported in the group that received service referrals vs. 4.8 (2.8) needs in the group that did not receive service referrals, *p* = 0.666.

**Table 3:** Comparison between receivers vs. non-receivers of 211 referrals

	No 211 referrals	Received 211 referrals	*p* value
Characteristic	*N* = 384	*N* = 98^a^	
Number of needs reported (SD)	4.8 (2.8)	4.7 (2.5)	0.666
Age in years (SD)	42.3 (14.2)	46.5 (17.0)	0.029
Gender			0.008
Female	189 (52.5%)	35 (37.2%)	
Male	171 (47.5%)	59 (62.8%)	
Ethnic background			0.042
Choose not to disclose	3 (0.8%)	0 (0.0%)	
Hispanic/Latino	74 (20.6%)	30 (32.3%)	
Not Hispanic/Latino	283 (78.6%)	63 (67.7%)	
Race			0.004
American Indian and Alaska Native	4 (1.1%)	2 (2.2%)	
Asian	2 (0.6%)	2 (2.2%)	
Black or African American	24 (6.7%)	8 (8.6%)	
Choose not to disclose	3 (0.8%)	0 (0.0%)	
Native Hawaiian and Pacific Islander	10 (2.8%)	0 (0.0%)	
Others	61 (16.9%)	30 (32.3%)	
White or Caucasian	256 (71.1%)	51 (54.8%)	

^a^Overall missing demographics data = 5.9%

Of the 1324 patients who indicated a social need, utilities were the most requested social need (668, 50.5%), followed by rent/mortgage (663, 50.1%) and clothing/furniture (655, 49.5%). Please see Table 4 for other social needs. The most common referral provided by 211 was for utility services assistance (29 participants, 29.6%), followed by rent payment assistance (26 participants, 26.5%) and food pantries (24 participants, 24.5%). Please see Table 5 for the six most common referrals provided.

**Table 4:** Frequency of social needs (*N* = 1324)

Social need	Yes	No	Prefer not to answer
Utilities	668 (50.5%)	632 (47.7%)	24 (1.8%)
Rent/mortgage	663 (50.1%)	638 (48.2%)	23 (1.7%)
Clothing/furniture	655 (49.5%)	647 (48.9%)	22 (1.7%)
Doctor/medical visit	605 (45.7%)	707 (53.4%)	12 (0.9%)
Food	594 (44.9%)	710 (53.6%)	20 (1.5%)
Employment	540 (40.8%)	761 (57.5%)	23 (1.7%)
Medication	486 (36.7%)	818 (61.8%)	20 (1.5%)
Housing	422 (31.9%)	888 (67.1%)	14 (1.1%)
Transportation	309 (23.3%)	1005 (75.9%)	10 (0.8%)
Childcare/eldercare	191 (14.4%)	1107 (83.6%)	26 (2.0%)

**Table 5:** Top six referral types

Referral type	*N* = 98
Utility service payment assistance	29 (29.6%)
Rent payment assistance	26 (26.5%)
Food pantries	24 (24.5%)
Low income/subsidized rental housing	16 (16.3%)
Navigator programs	8 (8.2%)
Food stamps/SNAP applications	8 (8.2%)

### Adoption

#### Staff observations and interviews

Themes from registration staff included (1) using professional intuition to decide whether, when, and who to screen; (2) determining if the chosen screener is the “right” tool for the ED system; and (3) questioning the usefulness of the screening as part of their staff role. There is also strong evidence supporting the hypothesis from our preliminary work that motivation (self-determination) plays a central role in adoption practices among front-line staff [13]. The staff who communicated that they felt like important members of the healthcare team, agents of change, or their role served a bigger purpose in the lives of patients—or intrinsic motivation—were more likely to integrate social needs screenings. In contrast, those communicating extrinsic motivation—feeling role expectations were prescribed by others—were likely to question their role in screening or to be skeptical of referrals to outside community resource agencies.

The vast majority of the registration staff reported leveraging their own “professional intuition” to deliver the social needs screener in what they saw was the more effective way. Such augmentations occurred during the introduction of the screener (the framing) or during the decision to screen (the value). Assumptions for these personal algorithms were based primarily on the staff perceptions of patient needs, specifically regarding insurance coverage, patient characteristics or demographics, current diagnoses, and/or a patients’ ability to engage with the screener. All such heuristics were noted as staff “judgment calls” occurring within moments of meeting the patient and prior to any clinical assessment or intake.

Observed approaches to screening ran the gamut, from completely confident to unsure, skeptical, and resentful. The staff felt strongly that, given their role and experience, that their professional intuition was fine-tuned enough to understand which patients may benefit (or not benefit) from the resultant services and to which the staff would alter their approach. About half of the staff participants also admitted to using their professional intuition to modify and/or take creative liberties “to make the screen [their] own.” Meaning, the staff would try to maintain each items’ integrity but tried to find more creative ways to ask or address the sensitive question line, etc. The staff who communicated higher professional intuition and intrinsic motivation utilized more modifications and took more creative liberty when delivering the screener. In terms of the professional role, there was little disagreement regarding the importance of screening for unmet social needs and its impact on the health of patients. One staff participant noted that “It is more than the patient; it is about the staff trusting that the information is useful.”

#### Patient community advisory group

Patient participants were mostly (*n* = 8, 80%) women and ranged in age from 25 to 60 years. The racial-ethnic composition of the group was 40% White, 20% Black, and 10% Asian/Pacific Islander; 20% of the group was identified as Hispanic/Latino. Educational preparation included 10% high school graduates, 40% some college, and 20% college graduates. Patient participants’ annual household incomes ranged from $10,000 to $75,000 or more, and the number of members in participants’ households ranged from 2 to 5.

Utilizing the “engagement studio” format, feedback revealed an important evolution of perspectives regarding social needs screening. Initially, there was a consensus on the value of the mission; each individual patient participant spoke of the importance in healthcare systems addressing their community’s unmet social needs. However, when exploring the screening process, critical feedback about the context and communication of screening and outreach emerged. The major themes—potential embarrassment, need for sincerity, and mitigating vulnerability—highlighted the important nuance when delivering social needs screening and concerns about the possible impact on intervention adoption.

Participant responses to the social needs screener were generally positive, with quotes of: “for me, I won’t have any problem,” “I’d be fine,” and simply “I’ve done these before.” The tone of initial conversations was casual, in which most participants confidently communicated that they would have no problem answering the social needs screening questions. Neutral comments were about the length of screening, if they would be feeling good enough to answer, or if language barriers might play into decisions regarding whether or not to respond to screening questions. Yet, quickly, participants moved their responses from themselves to thoughts of “others” and what others in their communities would do when asked to fill out the social needs screening. When they discussed what others would do, the responses transitioned to neutral (maybe they would do) as well as to negative (no, they would not do).

As participants continued to respond through the view of how “others” may feel, they identified negative comments noting that people could be embarrassed or that the screening could be seen as an invasion of personal privacy. The conversation explored more negative comments, divided out, and developed into the second theme of relatedness and vulnerability. After participants agreed they themselves would answer the screening questions, they were asked “who” should ask these questions. The theme of “I need to know YOU before I answer” developed from responses. The need for trust, comradery, and familiarity were described as participants stated they would disclose the personal information of social needs to those they felt “were genuine,” “were around the most,” and “who showed they cared.”

Questions related to how “showing you care” looks to these participants were offered as a probe, to which participants answered “eye contact,” “good personality,” “I liked her,” “you can just tell they care” were offered. One participant commented not “showing you care” is not expected of some staff such as registration staff offering “I know they are here about the business, the money.” An important discovery in this sub-theme was that, for participants to feel comfortable answering, they need/want to know the person who is asking the social needs screening questions, or feel a connection to them through a caring attitude, or a role with the expectation of caring for them. Specific examples offered by participants were nurses or social workers.

Vulnerability arose in the final theme from these patient-participants as they explored how they would feel when asked about their own social needs. Participants identified the main areas from which feelings of vulnerability stemmed, including (1) a patient who has needs may imply they are unable to provide for themselves, (2) referring for needs increases a patients’ exposure to outside agencies, and (3) they would not want these needs documented in the records that would “follow them around” in all clinical interactions. Participants reported particular community groups to which they would avoid disclosing their social needs due, in part, to their culture or past negative experiences applying for services; participants expressed concern that disclosing needs could increase a person’s vulnerability and open their exposure to systems outside of healthcare. One participant offered the example of revealing that childcare is a challenge may inadvertently expose a patient to action by child protective services. When prompted by the question about how screening results might be communicated to their healthcare providers, participants unanimously stated they would not want screening results in their medical record for fear that doing so would lead to being treated “differently” by healthcare providers. Further, they communicated concern that information may follow patients after personal circumstances change.

## Discussion

This study examined the reach and adoption of a Health Information Technology (HIT)-facilitated social needs screening and referral intervention; each arm represented a key aspect in the success of its design and implementation. Despite sparse evidence of technical and logistical barriers to universal social needs screening in the ED, fewer than 64% of targeted patients were screened, and 7% of those who communicated one or more social needs ultimately connected to services to address stated needs. Themes that emerged through qualitative data uncovered key, concrete messaging and training that may impact reach, adoption, and, ultimately, effectiveness of SDOH-social needs screening and referral efforts in clinical settings.

Our experiences of suboptimal intervention reach are not unique in a landscape of clinical interventions attempting to address patients’ social needs. A recent study by Hsu et al. [11] found that fewer than half of those participating in their SDOH screening and outreach efforts reported resolution of their screened social needs. These findings, combined with our own, suggest that barriers to SDOH intervention effectiveness likely exist both upstream and downstream from the point of service provider connections.

While our own intervention was developed with clinical implementation in mind (i.e., ease of delivery and existing staff and resources), our analysis of intervention reach and adoption clearly illustrates the impact of staff and patient-level factors act at multiple levels of the intervention, from decisions to approach, to complete screening, to accept outreach, and, while not measured in this study, to ultimately act on referrals. Also, similar to Hsu et al.’ [11] conclusion that patient collaboration, empathy, and positive regard are a product of interventionists acting as advocates, patients in our study communicated the need for relationship building and other signs the connote sincerity on behalf of those administering the intervention.

Overall, our study results may highlight a need to firmly ground SDOH interventions into the context of health behavior interventions, as influenced by concepts such as self-determination. Even in cases where a patient declines service referral, the work suggests there may be a benefit to the screeners’ inclusion in clinic workflow; the assessment still informs clinical interactions through a change in prescribed care and general knowledge regarding patient status. The fact that patients universally saw the impact of social needs and wanted their needs communicated to caring individuals suggest that, even when declining help in addressing social need, screening may still inform clinician-patient interactions. These findings are supported by the experiences of Tong et al. [12] who found that, despite reporting that assessing social needs is difficult and resource-intensive, clinicians also reported that knowing the patient had a social need changed care delivery and helped improve interactions with, and knowledge of, patients.

Complementary to the input of patients, our interview and observational data with staff demonstrate the need for additional, structured scripting and training related to presenting the screen’s question delivery, both in terms of overcoming time barriers but also in navigating staff-patient discomfort. Interventions to facilitate connectedness may also be useful, from the time of screening to ongoing engagement and problem-solving for those open to referrals. To overcome the discomfort and stigma of screenings, health systems seeking to implement social needs screening need to carefully consider strategies to better identify and promote staff’s intrinsic motivation and facilitate implementation readiness on the part of individual staff. Patient responses demonstrated how skills of communicating trust and commitment to building trust are a critical prerequisite for staff who are asking about patients’ unmet social needs. However, while allowing involved staff to pick and choose whether or how to implement the screen may facilitate staff autonomy, too much customization (i.e., leaving it up to staff to skip or modify the assessment) may, at best, threaten the fidelity of the screen and, at worst, result in patient “profiling” in a way that propagates and reinforces stigma around what patients already view as sensitive topics. As such, our findings suggest that developing clear methods for overtly addressing these areas related to the staff-patient experience of social needs screening, and evaluating their impact on reach and effectiveness may be a fruitful area for future research.

Facilitating intrinsic motivation and readiness could be further aided by more directive policies. We repeatedly found value in leveraging staff intuition about how/when to screen; they are most aware of the workflow’s opportunities and challenges to integration. To maximize adoption of any intervention, it is critical to integrate the priorities and context of key stakeholders, as well as maximize existing system infrastructure. This study argues that by formally recognizing these “human factors” of screening, we can better frame interventions to increase adoption and feasibility across clinical practice and roles. Finally, by understanding staff underlying motivation and autonomy, complex human variables, such as staff discomfort and stigma of social needs screening, can be operationalized and challenged through targeted education and workflow, thereby increasing relatedness and decreasing resistance.

One final, unambiguous, and strong concern common to both patients and staff participants was related to documenting social needs in the electronic health records. More specifically, patients voiced strong, unambiguous concerns over permanently documenting what they hope would are temporary life situations. This has important implications for efforts seeking to integrate SDOH-social needs data into clinical information systems: investments to insert and exchange these data in electronic health records are underway [8, 9, 36]. While clinicians may view social needs information as an important part of personalized care, patients also see this as different—and potentially lesser—care via patient profiling. Patients viewed screening only certain patient populations (e.g., by Medicare insurance) very unfavorably, and such efforts are even likely to undermine therapeutic relationships.

There are a number of limitations to this study. First, this intervention was conducted in one health sciences center emergency department; patients screened were relatively homogenous with respect to race/ethnicity with lower-than-expected completion in Spanish. Therefore, future efforts should purposively focus on the needs and experiences of non-English speakers during social needs screening and outreach, particularly as they likely face unique vulnerabilities. While we believe that our larger sample size of screeners helps protect from selection bias, our qualitative analysis is rooted in data from smaller groups of staff and patient participants. However, the fact that clear, consistent themes emerged from both of these samples and that themes can be clearly linked to the language of evidence-based behavioral frameworks such as self-determination theory increases our confidence that our findings can be generalized beyond our sample and should inform future research seeking to rigorously develop, refine, and test SDOH screening and referral processes with larger samples of those conducting screening and being screened.

While reach was limited to 20% of those who had needs and wished outreach, these numbers could have a marked impact on population health at scale. Further, our quantitative data are somewhat reassuring in that those ultimately engaging in the 211 outreach and receiving referrals are those who are more often members of underserved or underrepresented patient populations. However, our experiences demonstrate that universal screening for social needs in a busy ED setting, or likely any healthcare setting, may be difficult to maintain as a sustained and accurate practice without staff mechanisms for ongoing staff engagement and feedback mechanisms. Health systems seeking to address social determinants by integrating social needs assessments and community-based referrals must carefully consider policies that prepare staff by identifying and promoting their intrinsic motivation and facilitating readiness, and on methods for communicating sincerity and concern for patients’ individual circumstances. Additionally, because of the variability of resources and workflows across clinical settings, research should pursue a better understanding of what and where there is flexibility within the protocol for settings and staff to customize their approach.

## Conclusions

In contrast to the time and technology barriers commonly reported with SDOH screening in busy clinical settings, our study demonstrates that the reach and adoption of interventions aiming to address patients’ social needs are strongly influenced by key staff and patient factors related to stigma, privacy, and relatedness. Staff and patient perceptions of screenings suggest that, before scaling, implementation efforts ought to be accompanied by language that clearly communicates the universal nature and benefit of screening. Additionally, addressing the key pillars of self-determination theory—autonomy, competence, and relatedness—may serve as a useful framework for SDOH screening interventions. Future research and health system interventions should aim to test methods for connoting sensitivity and sincerity when screening and consider the ethical implications of documenting social needs in health records and subsequent impact on patient willingness to have needs addressed.

## Supplementary Information

**Additional file 1.** Interview guide – Question stems.

**Additional file 2.** Codebook for staff interviews.

**Additional file 3.** Pictorial representation and coding sample. Self-determination theory as a framework for mediating staff engagement in social needs screening interventions. Key: Bottom arm of the figure represents the ‘autonomy’ arm of self-determination theory as it relates to data. Right arm of the figure represents the ‘competence’ arm of self-determination theory as it relates to data. Left arm of the figure represents the ‘relatedness’ arm of self-determination theory as it relates to data.

## Data Availability

The datasets used and/or analyzed during the current study are available from the corresponding author on reasonable request.

## Abbreviations

211: United Way’s 2-1-1, https://uw.org/
ED: Emergency department
EHR: Electronic medical record
REDCap: Research Electronic Data Capture
SDOH: Social determinants of health
TRA: Theory of reasoned action
