# Supplementary material for: Integrating social determinants of health screening and referral during routine emergency department care: evaluation of reach and implementation challenges
Source: Implement Sci Commun. 2021 Oct 7;2:114. doi: 10.1186/s43058-021-00212-y (PMC8499465; doi:10.1186/s43058-021-00212-y)
Supplement: Supplementary file 1 — Additional file 1. Interview guide – Question stems. [file 43058_2021_212_MOESM1_ESM.docx]

**Additional file 1: Interview guide – Question stems**

| *Category* | *Item* | *Question stem & probe* | *Reference for category development* |
| --- | --- | --- | --- |
| Complex needs of patients | 1 | Screening for social needs is a new responsibility in your professional role in the ED. Sometimes it can be hard to fit in tasks/people into each encounter, and sometimes there are certain patients can make it difficult to complete the task.  Tell me about how it is going for the department? For you?  *Sub-questions:*   - What is working well or badly? - What can we do to improve?   Who are the people within the ED that you think should be involved in this process moving forward? | (Thomas-Henkel & Schulman, 2017; Andermann, 2018; Institute for Healthcare Improvement, 2019; Centers for Medicare and Medicaid Services, 2018) |
| Screening Ethics | 2 | Take me through the experience of screening a patient for social needs with patients in the ED?  *Sub-questions:*   - What type of patient or situation makes it hard to screen for social needs? - How standard (or not) is your approach in approaching patients to screen? - How do you introduce the screen? | (Garg, Boynton-Jarrett, & Dworkin, 2016; Andermann, 2018) |
| Complex needs of patients | 3 | Not screening a patient is a choice we make with every  What are some reasons you might not decide to screen a patient?  *Sub-questions:*   - Are there certain types of patients that you find difficult or intimidating to screen? - Are there certain areas of patient communication where you feel you untrained or unaware of how to proceed? - Do you change the framing of the screen depending on the patient? | (Thomas-Henkel & Schulman, 2017) |
| Complex need of patients | 4 | Tell me about your comfort level screening with patients who have co-occurring mental health concerns.  *Sub-question:*   - What is your current protocol around registering someone that you think has active mental health concerns? | (Thomas-Henkel & Schulman, 2017) |
| Quality Improvement | 5 | What would an ideal training for social needs screening be like for you?  *Sub-questions:*   - What would help minimize these barriers to your screening approach? - Which are the most critical pieces of information/training that you would at this time? | (Institute for Healthcare Improvement, 2019) |
| Caregiving | 6 | Who accompanies that patient to the hospital?   - Do you involve these people in the screening? If so, often do you involve them? How do you approach the screening process with them? - What role does a family member play in the process when there is a person with a medical condition that limits their ability to communicate such as with mental health issues? | (Sundar, 2018) |

**Additional file References**

Andermann, A. (2018). Screening for social determinants of health in clinical care: moving from the margins to the mainstream. *Public Health Reviews, 39*, 19. https://doi.org/10.1186/s40985-018-0094-7

Centers for Medicare and Medicaid Services (2018). *Accountable Health Communities Model.* Accessed on May 20, 2019 at <https://innovation.cms.gov/initiatives/ahcm/>

Garg, A., Boynton-Jarrett, R., & Dworkin, P. H. (2016). Avoiding the Unintended Consequences of Screening for Social Determinants of Health. JAMA. 2016;316(8):813–814. doi:10.1001/jama.2016.9282

Institute for Healthcare Improvement (2017). *QI Essentials Toolkit.* Boston, MA: ​Institute for Healthcare Improvement. Accessed on May 20, 2019 at <http://www.ihi.org/resources/Pages/Tools/Quality-Improvement-Essentials-Toolkit.aspx>

Sundar, S. (2018). Universal Screening for Social Needs in a Primary Care Clinic: A Quality Improvement Approach Using the Your Current Life Situation Survey. *The Permanente Journal, 22*, 18-089.

Thomas-Henkel, C. & Schulman, M (2017). *Screening for Social Determinants of Health in Populations with Complex Needs: Implementation Considerations*. Hamilton, New Jersey: Center for Health Care Strategies. Retrieved on May 20, 2019 at: https://www.chcs.org/ resource/screening-social-determinants-health-populations-complex-needs-implementation-considerations/
