# Supplementary material for: Integrating social determinants of health screening and referral during routine emergency department care: evaluation of reach and implementation challenges
Source: Implement Sci Commun. 2021 Oct 7;2:114. doi: 10.1186/s43058-021-00212-y (PMC8499465; doi:10.1186/s43058-021-00212-y)
Supplement: Supplementary file 2 — Additional file 2. Codebook for staff interviews. [file 43058_2021_212_MOESM2_ESM.docx]

| Additional file 2 | CODEBOOK FOR STAFF INTERVIEWS |
| --- | --- |
| *Code* | ***Comment*** |
| Acknowledging hierarchy of needs | When a staff member deems the screening as a lower clinical or administrative priority in the ED |
| Agreeing with screening purpose | Patient personally agrees the purpose of the screen and can reiterate its mission. Related code: Internalizing the screen’s mission (also see self-determination diagram for levels of engagement) |
| Appropriateness of screening team | There are questions or concerns voiced by the staff regarding the appropriateness of the registration staff being the “right” group to own this workflow. |
| Assuming needs | Staff discussing their assumptions being made regarding patient needs and whether or not a patient should receive the screener. |
| Being skeptical about hospital integration strategy | Underlying doubt or skepticism from the interviewee about the greater purpose/meaning of the 211 and ED collaboration (ie. other agendas at play) |
| Describing caregiver approach |  |
| Describing caregiver responses | Description of the role that caregivers play in the screening process of a patient |
| Describing patient groups per staff consensus | Acknowledging the existence of patient cohorts and definitely/nicknames. |
| Developing professional intuition | Discussion of how the staff member was able to develop professional intuition and experience regarding empiric algorithms of who and who not to screen for social needs |
| Disagreement among multiple caregivers | When multiple caregivers in the room and the group’s answers vary regarding the patient’s unmet social needs. Or, there are different perspectives/answers about screening participation. |
| Discussing caregivers role in screening | Mentioning the role that caregivers play in screening |
| Discussing comfort with screening mental health patients | Participant discusses their level of comfort in screening those ED patients with co-occurring mental diagnoses or needs. |
| Discussing personal comfort level of screening patients | Personal assessment of their staff’s  comfort level in screening patients |
| Discussing the need for extra training | Study participants addressing the need (or lack thereof) for additional training regarding skills or explanations re: screening tool and need |
| Enjoying patient stories on newsletter | Positive response to weekly ED-211 newsletter stories where the team reports back deidentified case studies of successful resource connections for patients. |
| Exemplar Quote -- Screening decisions | "I am comfortable asking it, if it is relevant.” — possible theme emerging |
| Feeling misunderstood in role or capacity | Staff reports feeling shutdown or underappreciated in their role with the screen. |
| Feeling negative about being a screener | Staff reports resentment or negative feelings toward being put in the position of being the screener |
| Giving the option for self vs. staff administration of screen | Study participant reports that he/she/they gives the patient/caregiver an option in having the screener read out-loud to them in the patient room or have the patient/caregiver fill it our privately on the iPad |
| Giving the option to participate | Staff explicitly offers patient/caregiver the choice of participating in the 211-ED screen |
| Improving screening | Ideas for how to improve the screen itself (not the process)Related code: Improving |
| Interviewee shutting down | Interviewer notices the participant shutting down in the conversation due to shortened response style or interpersonal cues |
| Introducing screen | The wording used to introduce the screener to a patient/caregiver, as reported by staff |
| Judging ability through intuition, experience | When a staff member uses professional intuition or experience (empiric) to decide which patients get screened and when. |
| Making no changes or modifications to screen | Staff reports not making any changes to screening protocol. No personal edits, modifications, or creative liberties  explicitly mentioned. |
| Observing patient trepidation | Interviewee witnesses and reports patient’s feeling of trepidation regarding screen parts |
| Patient as primary concern | Regarding caregiver involvement, the staff reports patient as primary screening target/ |
| Perceiving comfort with skillset, but has boundaries | This statement is about staff who believe that they have the required skills and comfort level to successfully screen patients, but then follow up their answer with a qualifying statement. Perhaps admitting boundaries to their competence. Related code: “Qualifying statement re: abilities…" |
| Perceiving time as a barrier to screening patients | Interviewee reports time as a barrier to integration of social needs screener. |
| Preferring screen is performed by another team | Participant makes a reference regarding who should be performing the ED screening |
| Qualifying statement re: abilities, intuition, or experience | Qualifying statement offered by interviewee after discussing that he/she/they have the required skills and comfort level to successfully screen patients. Perhaps admitting boundaries to their competence. Related code: “Perceiving comfort with skillset.." |
| Questioning line unclear -- rent | Reports of patient uncertainty or reluctance to answer the rent questions. Could be related to its ultimate purpose, question framing, or need for such information. |
| Questioning why hospital cannot provide their own resources | Interviewee has questions of why/how the hospital cannot already address unmet social needs. |
| Relating to survey fidelity | Having to do with whether or not survey protocol was followed and instrument fidelity. |
| Repeating answer | Participant notes to interviewer that he/she/they have already answered the questions asked. |
| Responding to study consent | Participant response to study protocol and consent process |
| Rethinking question line here | Reserved for internal use. This question may need to be reworded, reframed, or perhaps tossed altogether. They may not know what they don’t know on the topic. Additionally, it may not be their role or priority to be a part of this extended QI project. |
| Reviewing personal screening protocol | Discussing one’s personal experience in administering the screener (i.e. how to introduce it, what they do, etc.) |
| Security guards as gatekeepers | Reporting that the ED’s security guards are also play a role in dictating which patients should and should not be screened |
| Skipping patients -- "gold pod" | Staff reporting skipping patients types based upon location of patient in the “gold pod” (i.e. psych beds) |
| Skipping patients -- d/t combative, agitated | Staff reporting skipping patients types based upon reports of patient being combative or agitated. Baseline safety concerns. |
| Skipping patients -- d/t intuition | Staff reporting skipping patients types based upon professional intuition. |
| Skipping screen -- d/t Medicare/Medicaid coverage | Patients are “skipped” due to Medicaid/Medicare coverage, under the assumption that someone else if performing the task. |
| Skipping screen-- d/t level of consciousness | When an interviewee reports that he/she/they skip patients d/t level of consciousness (ie. Glasgow) or ability to physically participate in the screening. |
| Training opportunities | Participant references the need (or lack thereof) training experiences pertaining to social need screening in the ED |
| Understanding purpose of screening as a public health intervention | Interviewee reports understanding the mission/purpose of the social needs screener, in terms of its public health ramifications and patient health outcomes. |
| Using professional intuition about screening decisions | Study participant references using his/her/their intuition and experience to make decisions about who should and should not be screened. |
