# Supplementary material for: Integrating social determinants of health screening and referral during routine emergency department care: evaluation of reach and implementation challenges
Source: Implement Sci Commun. 2021 Oct 7;2:114. doi: 10.1186/s43058-021-00212-y (PMC8499465; doi:10.1186/s43058-021-00212-y)
Supplement: Supplementary file 3 — Additional file 3. Pictorial representation and coding sample. Self-determination theory as a framework for mediating staff engagement in social needs screening interventions. Key: Bottom arm of the figure represents the ‘autonomy’ arm of self-determination theory as it relates to data. Right arm of the figure represents the ‘competence’ arm of self-determination theory as it relates to data. Left arm of the figure represents the ‘relatedness’ arm of self-determination theory as it relates to data. [file 43058_2021_212_MOESM3_ESM.docx]

**Additional file 3: Pictorial representation and coding sample**

*Title: Self-determination theory as a framework for mediating staff engagement in social needs screening interventions*


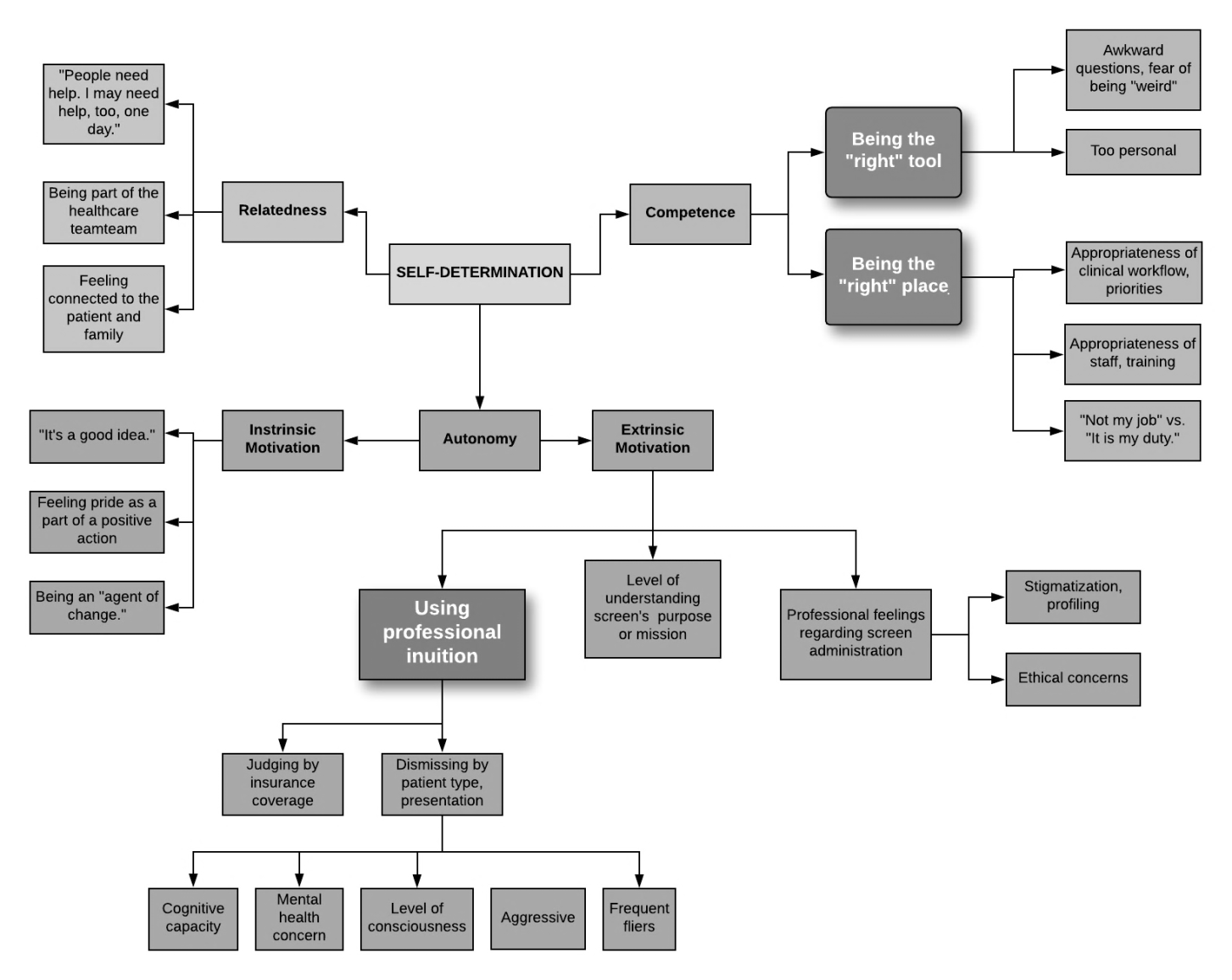


| **KEY** |
| --- |
| *Bottom arm of the figure represents the ‘autonomy’ arm of self-determination theory as it relates to data*  *Right arm of the figure represents the ‘competence’ arm of self-determination theory as it relates to data*  *Left arm of the figure represents the ‘relatedness’ arm of self-determination theory as it relates to data* |
